# Cost and value in liver disease guidelines: 2011–2022

**DOI:** 10.1097/HC9.0000000000000001

**Published:** 2023-01-03

**Authors:** Elizabeth S. Aby, Alyson Kaplan, Nneka N. Ufere

**Affiliations:** 1 Division of Gastroenterology, Department of Medicine, Hepatology, and Nutrition, University of Minnesota, Minneapolis, Minnesota, USA; 2 Department of Gastroenterology and Hepatology, New York Presbyterian, Weill Cornell Medicine, New York, New York, USA; 3 Liver Center, Gastrointestinal Unit, Massachusetts General Hospital, Boston, Massachusetts, USA

## Abstract

Chronic liver disease (CLD) is associated with rising health care utilization and cost. We aimed to describe the frequency of cost/value (C/V) statements in CLD-related clinical guidance documents (CGDs).

**Methods:**

CGD with a focus on CLD published between January 2011 and February 2022 from 3 US societies [Association for the Study of Liver Diseases (AASLD), American College of Gastroenterology (ACG), and American Gastroenterological Association (AGA)] were analyzed.

**Findings:**

Forty-five CGDs were identified. Eighty of 1334 guidance statements were C/V statements (6%). Only 1.1% reported patient-level costs and none reported out-of-pocket costs. Despite the increased importance of incorporating cost and value into care, the proportion of C/V statements in CGDs related to liver disease is low.

## INTRODUCTION

The economic burden of liver disease in the United States is rising. In 2016 alone, health care spending for patients with chronic liver disease (CLD) exceeded $32 billion, or 1.2% of total US health care expenditures.^1^ As costs of liver disease care rise, there is a growing need for more explicit and transparent assessments of the financial burden of liver disease on patients.^2^

Professional society clinical guidance documents (CGD) are created to inform clinicians of best clinical practices and to encourage shared decision making with patients to promote the delivery of high-value care. While cost-effectiveness data are often considered in the creation of CGD as a part of the Grading of Recommendations, Assessment, Development, and Evaluation process, it is unclear how patient-level costs are incorporated into CGD. In this study, we aimed to quantify and categorize the frequency of cost/value (C/V) statements in liver disease related CGD from three US gastroenterology and hepatology professional societies published between 2011 and 2022: the American Association for the Study of Liver Diseases (AASLD), the American Gastroenterological Association (AGA), and the American College of Gastroenterology (ACG).

We identified a total of 45 CGD from AASLD (n=22), AGA (n=14), and ACG (n=9). In total, 21 (46.7%) were clinical practice guidelines (CPG), 23 (51.1%) were expert consensus documents (ECD), and 1 (2.2%) had both ECD and CPG components. All CGDs included in the analyses are described in Table 1. Cost and value considerations were classified based on protocols adapted from prior studies.^3^,^4^ See Supplementary Material, http://links.lww.com/HC9/A18 for further description of the methodology.

**TABLE 1:** Documents included in the analysis

Document Number	Clinical Guidance Document Title	Society	Year	Type of Document	Total Number Statements	Percentage of Patient-Level Cost Statements	Percentage of C/V Statements	Examples of C/V Statements
1	American Gastroenterological Association Institute Guideline on the Prevention and Treatment of Hepatitis B Virus Reactivation During Immunosuppressive Drug Therapy	AGA	2015	CPG	9	22	56	“Cost-effectiveness studies of HBV screening in patients with cancer have shown that screening is cost-beneficial in patients with non-Hodgkin lymphoma slated to receive rituximab and may be cost-effective in patients with breast cancer slated to receive adjuvant chemotherapy if HBV infection is prevalent”
2	AGA Clinical Practice Update on Screening and Surveillance for Hepatocellular Carcinoma in Patients With Nonalcoholic Fatty Liver Disease: Expert Review	AGA	2020	ECD	8	13	50	“In general, the incidence rate of HCC in NAFLD cirrhosis is estimated to be >1.5% per year and, therefore, screening for HCC in this group is justifiable, based on cost-effectiveness considerations”
3	AASLD Guidelines for Treatment of Chronic Hepatitis B	AASLD	2015	CPG	15	20	33	Treatment of Adults with Immune Tolerate Chronic Hepatitis B: “Given the lack of evidence of benefit to those with ALT <ULN (indicative of immune-tolerant CHB), the potential harms of finite (or longer) antiviral therapy, including cost, antiviral drug side effects, and development of resistance (with NAs), outweigh benefits”
4	AGA Clinical Practice Update on Interaction between Oral Direct-Acting Antivirals for Chronic Hepatitis C Infection and Hepatocellular Carcinoma: Expert Review	AGA	2019	ECD	12	8	25	“The absolute risk of HCC remains high in patients with cirrhosis at the time of SVR with DAA, with the overall annual risk ranging between 1.8% and 2.5% in different studies. These estimates reached or exceeded the cutoffs (0.8%–1.5% per year) beyond which HCC surveillance becomes cost-effective”
5	AGA Clinical Practice Guideline on the Management of Coagulation Disorders in Patients With Cirrhosis	AGA	2021	CPG	8	13	25	“Blood product transfusion can be lifesaving, but the risk of exacerbating portal hypertension from volume expansion, bacterial and viral contamination, immunologic complications, and cost make it imperative to restrict their use whenever possible”
6	AGA Clinical Practice Update on Palliative Care Management in Cirrhosis: Expert Review	AGA	2021	ECD	10	0	20	“On the basis of the median national wage for a home health aide, the annual costs of provision of care to older adults with cirrhosis in this study were estimated to be more than double those for an age-matched comparison group (for 2009, $4700 per person with cirrhosis compared with $2100 without cirrhosis)… The financial implications of caregivers of patients with cirrhosis are profound if evaluated in a comprehensive fashion… Caregiver burden is common, especially in patients with decompensated disease. It has physical, mental, and financial consequences…Repeated hospital admissions, alcohol as etiology, and lower socioeconomic status were independent predictors of caregiver burden”
7	AGA Institute Guideline on the Role of Elastography in the Evaluation of Liver Fibrosis	AGA	2017	CPG	11	0	18	“We did not factor in cost-effectiveness of vibration-controlled transient elastography vs. serum-based fibrosis markers, given rapidly changing prices of antiviral therapy, which may offset cost-to-benefit assessments”
8	American Gastroenterological Association Institute Guidelines for the Diagnosis and Management of Acute Liver Failure	AGA	2017	CPG	11	9	18	For herpes simplex virus hepatitis: “There is little downside to treatment with acyclovir from cost or adverse event standpoints”
9	Diagnosis and Management of Hemochromatosis: 2011 Practice Guideline by the American Association for the Study of Liver Diseases	AASLD	2011	CPG	18	6	16	“Economic models that have included genetic testing have suggested that population screening for hereditary hemochromatosis would be effective if only 20% of patients developed life-threatening complications; and the costs of investigating false positive iron tests in a screening program were considered significant”
10	AASLD Guidelines for the Treatment of Hepatocellular Carcinoma	AASLD	2018	CPG	15	7	13	“Biopsy is expensive, may cause anxiety or pain, and has a risk of complications, including tumor track seeding and bleeding”
11	Hepatic Encephalopathy in Chronic Liver Disease: 2014 Practice Guideline by the American Association for the Study of Liver Diseases and the European Association for the Study of the Liver	AASLD	2014	CPG	30	7	13	“The occurrence of MHE and CHE in patients with CLD seems to be as high as 50%, so, ideally, every patient at risk should be tested. However, this strategy may be costly, and the consequences of the screening procedure are not always clear and treatment is not always recommended”
12	Malnutrition, Frailty, and Sarcopenia in Patients With Cirrhosis: 2021 Practice Guidance by the American Association for the Study of Liver Diseases	AASLD	2021	ECD	38	3	13	“Given the ease, low cost, and repeatability of frailty metrics, we recommend routine assessment of frailty using a standardized tool—from which there are many to choose”
13	ACG Clinical Guideline: Hereditary Hemochromatosis	ACG	2019	CPG	10	0	10	“One study estimated that the cost of screening children of C282Y homozygous patients with hereditary hemochromatosis could be reduced by 39% by genotyping the spouses of the probands. A separate study found that screening the siblings of patients was cost saving, and the use of HFE gene testing was generally more cost effective than serum iron studies. Cost savings were realized by avoiding repeat testing among individuals not genetically at risk of iron overload”
14	American Gastroenterological Association Institute Clinical Practice Update—Expert Review: Care of Patients Who Have Achieved a Sustained Virologic Response After Antiviral Therapy for Chronic Hepatitis C Infection	AGA	2017	ECD	11	9	9	“This recommendation is based on considerations of cost-effectiveness and the historical use of ultrasound in studies that have shown an impact on outcomes of early detection of HCC. Despite its greater cost than computed tomography, MRI has the advantage of avoiding exposure to ionizing radiation”
15	ACG Clinical Guideline: Disorders of the Hepatic and Mesenteric Circulation	ACG	2020	CPG	23	0	9	“Doppler ultrasonography is noninvasive, low cost, and correlates well with pathology and venogram findings”
16	AGA Clinical Practice Update: Coagulation in Cirrhosis	AGA	2019	ECD	12	0	8	“Despite this, platelets are commonly transfused in patients with cirrhosis and thrombocytopenia before invasive procedures. This strategy poses some risk to patients, given the short half-life of the transfusions, cost, and the possibility of alloimmunization and other adverse reactions”
17	Diagnosis, Staging, and Management of Hepatocellular Carcinoma: 2018 Practice Guidance by the American Association for the Study of Liver Diseases	AASLD	2018	ECD	24	0	8	“Despite their high diagnostic performance, cross-sectional, multiphase, contrast-enhanced computed tomography (CT) or magnetic resonance imaging (MRI) are not recommended for HCC surveillance given the paucity of data on their efficacy and cost-effectiveness”
18	AGA Clinical Practice Update on Management of Bleeding Gastric Varices: Expert Review	AGA	2021	ECD	12	0	8	“While direct comparisons of TIPS with endoscopic cyanoacrylate injection suggest similar efficacy in initial hemostasis with slightly better rates of long-term rebleeding with TIPS, they also suggest added costs and increased complications (encephalopathy) with TIPS placement”
19	Portal Hypertensive Bleeding in Cirrhosis: Risk Stratification, Diagnosis, and Management: 2016 Practice Guidance by the American Association for the Study of Liver Diseases	AASLD	2016	ECD	51	0	8	“Ultrasound provides safe and inexpensive imaging evidence of morphological abnormalities associated with cirrhosis and portal hypertension”
20	Hepatitis C Guidance 2019 Update: American Association for the Study of Liver Diseases Infectious Diseases Society of America Recommendations for Testing, Managing, and Treating Hepatitis C Virus Infection	AASLD	2020	CPG	28	0	7	“Modeling studies support treatment of acute HCV infection, demonstrating cost-effectiveness assuming high treatment efficacy with a relatively short treatment duration—requisite conditions that currently exist”
21	AASLD Practice Guidance: Palliative care and symptom-based management in decompensated cirrhosis	AASLD	2022	ECD	66	2	6	“Assessing financial burden is also important for patients with decompensated cirrhosis, who often report high rates of cost-related nonadherence to medications and food insecurity”
22	The Diagnosis and Management of Nonalcoholic Fatty Liver Disease: Practice Guideline by the American Association for the Study of Liver Diseases, American College of Gastroenterology, and the American Gastroenterological Association	AASLD	2012	CPG	36	0	6	“Liver biopsy remains the gold standard for characterizing liver histology in patients with NAFLD. However, it is expensive and carries some morbidity and very rare mortality risk”
23	ACG Clinical Guideline: The Diagnosis and Management of Focal Liver Lesions	ACG	2014	CPG	38	0	5	“Two randomized controlled trials and a meta-analysis based on these trials reported that PAIR with adjunct antihelminthic chemotherapy is as effective as open surgical drainage with fewer complications and lower cost”
24	Update on Prevention, Diagnosis, and Treatment of Chronic Hepatitis B: AASLD 2018 Hepatitis B Guidance	AASLD	2018	ECD	76	1	5	“AFP alone is not recommended except in those circumstances where United States is unavailable or cost is an issue. HCC surveillance is considered cost-effective if the annual risk of HCC is >0.2% per year”
25	Primary Biliary Cholangitis: 2018 Practice Guidance from the American Association for the Study of Liver Diseases	AASLD	2018	ECD	21	1	5	“The dose of UDCA is important. A study comparing three different doses of UDCA showed that a dose of 13–15 mg/kg/day appeared superior to either a lower dose of 5–7 mg/kg/day or a higher dose of 23–25 mg/kg/day in biochemical responses and cost”
26	The Diagnosis And Management of Nonalcoholic Fatty Liver Disease: Practice Guidance from the American Association for the Study of Liver Diseases	AASLD	2018	ECD	43	0	5	“A recent, cost-effective analysis using a Markov model suggested that screening for NASH in individuals with diabetes is not cost-effective at present, because of disutility associated with available treatment”
27	Diagnosis and Treatment of Alcohol-Associated Liver Diseases: 2019 Practice Guidance From the American Association for the Study of Liver Diseases	AASLD	2020	ECD	22	0	5	“Ethyl glucuronide: Costly, requires significant hair sample, limited availability. More costly than urine Ethyl glucuronide”
28	ACG Clinical Guideline: Primary Sclerosing Cholangitis	ACG	2015	CPG	24	0	4	“MRCP is noninvasive, less expensive than ERCP, and has no associated risk of pancreatitis”
29	Diagnosis and Management of Autoimmune Hepatitis in Adults and Children: 2019 Practice Guidance and Guidelines From the American Association for the Study of Liver Diseases	AASLD	2020	ECD/CPG	52	2	4	“Absent or near-absent TPMT activity occurs in only 0.3%–0.5% of the normal population, but the possibility of preventing severe bone marrow toxicity may warrant its use without an analysis of cost-effectiveness”
30	Acute-on-Chronic Liver Failure Clinical Guidelines	ACG	2022	CPG	27	0	4	“Given the expense, logistic challenges of setting up infusions and potential for causing pulmonary edema, the effectiveness of IV albumin in conditions other than SBP and postparacentesis circulatory dysfunction needs more study”
31	Vascular Liver Disorders, Portal Vein Thrombosis, and Procedural Bleeding in Patients With Liver Disease: 2020 Practice Guidance by the American Association for the Study of Liver Diseases	AASLD	2020	ECD	58	0	3	Defibrotide for sinusoidal obstructive syndrome: “In the past, some authorities have recommended it for prophylaxis in high-risk cases, but the benefit of this approach is unproven, and the acquisition costs for the drug are very high”
32	AASLD Position Paper: The Management of Acute Liver Failure: Update 2011	AASLD	2011	ECD	48	0	2	“Important barriers to the routine use of rFVIIa remain, however, including its very high cost, and reports of serious thromboembolism in patients with acute liver failure (myocardial infarction and portal vein thrombosis)”
33	Evaluation for Liver Transplantation in Adults: 2013 Practice Guideline by the AASLD and the American Society of Transplantation	AASLD	2013	CPG	56	2	2	“Given today’s complexities of insurance for medical care, it is also necessary to ensure that a potential recipient will have adequate posttransplant medication coverage”
34	Diagnosis, Evaluation, and Management of Ascites, Spontaneous Bacterial Peritonitis and Hepatorenal Syndrome: 2021 Practice Guidance by the American Association for the Study of Liver Diseases	AASLD	2021	ECD	60	0	2	“Thus, the discrepant results between the two trials point to the need for further studies to address the role of albumin as well as cost-effectiveness in the management of ascites”
35	Long-Term Management of the Successful Adult Liver Transplant: 2012 Practice Guideline by AASLD and the American Society of Transplantation	AASLD	2012	CPG	98	0	1	“The individualization of prophylactic combination therapy can be undertaken on the basis of pretransplant clinical and virological characteristics. For example, low-dose intramuscular HBIG is much less expensive and avoids painful side effects associated with intravenous HBIG”
36	Reproductive Health and Liver Disease: Practice Guidance by the American Association for the Study of Liver Diseases	AASLD	2021	ECD	103	0	1	Hepatitis C virus: “Limitations of risk-based screening are well recognized and universal screening is recommended and cost-effective”
37	AGA Clinical Practice Update on Diagnosis and Management of Immune Checkpoint Inhibitor Colitis and Hepatitis: Expert Review	AGA	2021	ECD	3	0	0	NA
38	AGA Clinical Practice Update on Surveillance for Hepatobiliary Cancers in Patients With Primary Sclerosing Cholangitis: Expert Review	AGA	2019	ECD	8	0	0	NA
39	ACG Clinical Guideline: Alcoholic Liver Disease	ACG	2017	CPG	9	0	0	NA
40	AGA Clinical Practice Update on Bariatric Surgery in Cirrhosis: Expert Review	AGA	2021	ECD	10	0	0	NA
41	Medical Management of Severe Alcoholic Hepatitis: Expert Review from the Clinical Practice Updates Committee of the AGA Institute	AGA	2017	ECD	12	0	0	NA
42	ACG Clinical Guideline: Evaluation of Abnormal Liver Chemistries	ACG	2017	CPG	19	0	0	NA
43	ACG Clinical Guideline: Diagnosis and Management of Idiosyncratic Drug-Induced Liver Injury	ACG	2021	CPG	24	0	0	NA
44	Hepatitis C Guidance 2018 Update: AASLD-IDSA Recommendations for Testing, Managing, and Treating Hepatitis C Virus Infection	AASLD	2018	ECD	29	0	0	NA
45	ACG Clinical Guideline: Liver Disease and Pregnancy	ACG	2016	CPG	36	0	0	NA

Abbreviations: AASLD, American Association for the Study of Liver Diseases; ACG, American College of Gastroenterology; AFP, alpha-feto protein; AGA, American Gastroenterological Association; C/V, cost value; CHE, cover hepatic encephalopathy; DAA, direct-acting antiviral; ERCP, endoscopic retrograde cholangio pancreatography; HBIG, Hepatitis B Immune Globulin; HBV, Hepatitis B Virus; HCC, hepatocellular carcinoma; HFE, hereditary hemochromatosis type 1; IDSA, Infectious Diseases Society of America; MHE, minimal hepatic encephalopathy; MRCP, magnetic resonance cholangio pancreatography; PAIR, puncture, aspiration, injection, and re-aspiration; rFVIIa, Recombinant Activated Factor VIIa; SBP, spontaneous bacterial peritonitis; SVR, systemic vascular resistance; UDCA, ursodeoxycholic acid.

In the 45 CGD, a total of 1334 guidance statements were made, of which 80 (6.0%) were C/V statements [AASLD (n=50), AGA (n=23), and ACG (n=7)]. Nine (20.0%) CGD contained no C/V statements. The median number of C/V statements was 1 (interquartile range: 1–2.3) in ECD and 2 (interquartile range: 1–2) in CPG. A similar proportion of the 80 C/V statements were found in ECD (n=41, 51.3%) compared to CPG (n=39, 48.7%). The AGA had the highest percentage of C/V statements out of the total number of guidance statements (16.8%) compared to the AASLD (5.3%) and the ACG (3.3%) (*P*<0.001) (Supplemental Figure 1, http://links.lww.com/HC9/A16).

Of the 80 C/V statements, the majority (85%) used costs implicitly to support the recommendation, whereas 12.5% explicitly stated that cost was integrated into the recommendation. One statement intentionally excluded cost in its considerations. When comparing CGD from 2011 to 2016 versus 2017 to 2022, there was no difference in the proportion of C/V statements between these time periods (6.3% vs. 5.8%, *p*=0.7).

Of the 80 C/V statements, 15 (18.8%) highlighted the economic impact of liver disease or its management (Supplemental Figure 2, http://links.lww.com/HC9/A17). An example of this was in the AGA Clinical Update on the Interaction between oral direct-acting antiviral agents for chronic hepatitis C and HCC: “In patients with cirrhosis, compared with no surveillance, semi-annual HCC surveillance every 6 months for up to 15 years was cost-effective using willingness-to-pay threshold of $100,000/quality-adjusted life-year.”^5^ Five statements (6.3%) advocated for C/V issues in clinical care, such as in the ACG acute-o- chronic liver failure clinical guidelines: “Given the expense, logistic challenges of setting up infusions and potential for causing pulmonary edema, the effectiveness of IV albumin in conditions other than spontaneous bacterial peritonitis and postparacentesis circulatory dysfunction needs more study.”^6^ Eight statements (10.0%) reported gaps in C/V evidence in hepatology, such as AASLD’s palliative care and symptom-based management in decompensated cirrhosis practice guidelines: “Assessing financial burden is also important for patients with decompensated cirrhosis, who often report high rates of cost-related nonadherence to medications and food insecurity.”^7^ Lastly, 52 (65.0%) C/V statements justified guidance recommendations, such as the AASLD primary biliary cholangitis practice guidelines: “The dose of UDCA is important. A study comparing three different doses of UDCA showed that a dose of 13 to 15 mg/kg/day appeared superior…in biochemical responses and cost.”^8^ Statement categorization did not differ by clinical CGD type (*p*=0.3) or time period (*p*=0.3).

Of the 52 C/V statements that justified guidance recommendations, 37 (71.1%) focused on societal-level costs and 15 (28.9%) focused on patient-level costs. Of these 52 C/V statements justifying guidance recommendations, 34 (65.4%) recommended use of an intervention due to its incremental benefit justifying the additional cost (n=20), equal effectiveness at lower cost (n=8), or to reduce future costs (n=6). For example, the AGA clinical practice update on screening and surveillance for HCC in patients with NAFLD justified screening individuals with NAFLD for HCC because the incidence of HCC in this population was estimated to be >1.5%.^9^

A total of 18 (34.6%) C/V statements discouraged the use of an intervention either due to an uncertain cost-to-benefit ratio (n=9) or because its incremental benefit did not justify the additional cost (n=9). For example, in the AASLD guidance statement on malnutrition, frailty, and sarcopenia, routine use of computed tomography was not recommended in the assessment of muscle mass in patients with cirrhosis due to cost as well as exposure to ionizing radiation.^10^ In the AASLD guidance statement on the diagnosis and management of NAFLD, routine screening for nonalcoholic steatohepatitis in individuals with diabetes was not felt to be cost-effective because of disutility associated with available treatment and thus was not recommended.^11^ No C/V statements reported estimated out-of-pocket costs for recommended therapies.

Despite the increased recognition of the rising economic and financial burden of liver disease care, our analysis demonstrates that C/V assessments were present in only 6% of guidance statements in CGD from the AASLD, AGA, and ACG. The majority of available C/V assessments justified guidance recommendations based on cost-effectiveness analyses that supported the use of an intervention despite the additional cost. Notably, only 15 (1.1%) out of a total of 1334 guidance statements reported patient-level costs of care and none explicitly reported out-of-pocket costs.

With limited patient-level data available on costs of liver disease care, it becomes challenging for clinicians to support patients with liver disease in their medical decision making based on their capacity to pay for the available treatment options. Prior work has shown that while clinicians acknowledge the responsibility to have cost discussions with patients, very few report a firm understanding of the out-of-pocket costs of health care. This deficit has significant implications as diagnostic, surveillance, and therapeutic management options in liver disease care continue to expand, such as for patients with decompensated cirrhosis and HCC.^12^ Nationally, over 1 in 4 patients with CLD experiences financial hardship from medical bills, which is associated with increased cost-related medication nonadherence and health care resource utilization.^13^ In addition, cost-related nonadherence has been shown to be associated with maladaptive cost-reducing behaviors.^14^ Given the prevalence of financial hardship for those with CLD, practitioners should consider screening for financial burden with questions such as, “are you having difficult with paying for your medical care?”^7^ If concerns are expressed, early social work and pharmacy support services should be engaged.

The development of a systematic approach to grade patient-level costs may guide the incorporation of C/V assessments into CGD, such as the use of value frameworks by national cardiology and oncology professional societies. Including granular price transparency data in CGD may also help clinicians to guide patients when choosing options that are feasible and effective based on potential financial constraints. For example, the American Diabetes Association CGD on pharmacologic management for glycemic treatment reports the median monthly costs for glucose-lowering agents.^15^ For physicians, it is important to keep in mind that the cost of medications can also exacerbate disparities. Within the hepatology literature, researchers have found that rifaximin, an expensive nongeneric medication, has been prescribed less frequently to Black and Hispanic patients. Being mindful about how cost is incorporated into individual patient decision making is important going forward.^16^

The main limitations of this study are the focus on CGD from US-based societies and the qualitative approach to the analysis. We acknowledge the complexity of integrating C/V statements into CGD due to limited high-quality evidence regarding value-based care in hepatology, shifting reimbursement structures, distinct value assessments between organizations and payors, and variations in market prices.

In summary, the proportion of C/V statements in AASLD, AGA, and ACG CGD related to liver disease is low and even lower for patient-level costs. Given the rising health care costs for liver disease, future studies are needed to better understand the financial burden, cost, and value of liver-related diagnostics and therapeutics. Now is the time for gastroenterology and hepatology professional societies to take on the challenge of integrating patient-level costs into guidance statements to improve the financial burden of patients with liver disease.

## Supplementary Material
