# Supplementary figures and images for: Cost and value in liver disease guidelines: 2011–2022
Source: Hepatol Commun. 2023 Jan 3;7(1):e0001. doi: 10.1097/HC9.0000000000000001 (PMC9827964; doi:10.1097/HC9.0000000000000001)

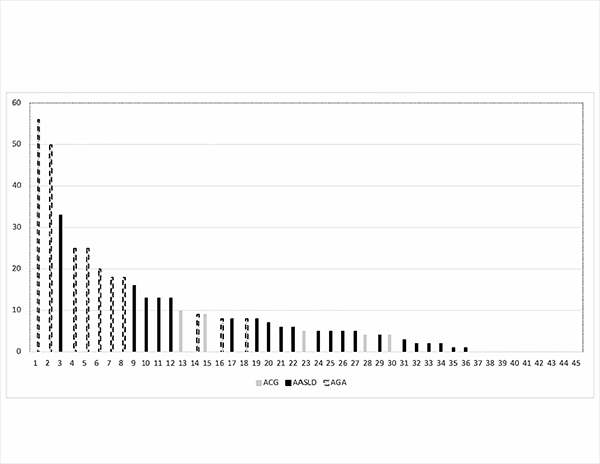

Supplement: Supplementary file 1 [file hc9-7-e0001-s001.tif]

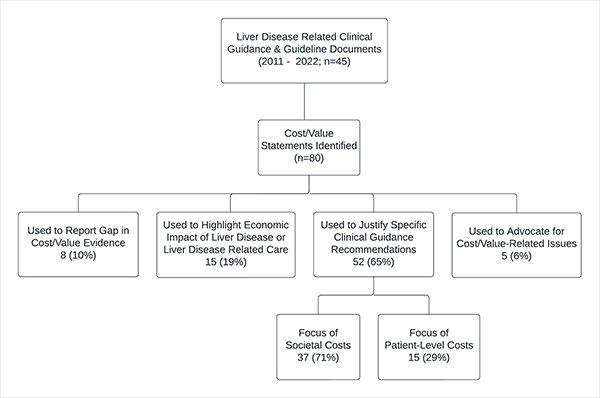

Supplement: Supplementary file 2 [file hc9-7-e0001-s002.tif]
