# Supplementary material for: Cost and value in liver disease guidelines: 2011–2022
Source: Hepatol Commun. 2023 Jan 3;7(1):e0001. doi: 10.1097/HC9.0000000000000001 (PMC9827964; doi:10.1097/HC9.0000000000000001)
Supplement: Supplementary file 3 [file hc9-7-e0001-s003.docx]

**Supplementary Material**

**Methods**

Clinical guidance documents (CGD) with a focus on liver disease published between January 2011 and February 2022 from the major gastroenterology and hepatology societies in the United States – American Association for the Study of Liver Diseases (AASLD), American College of Gastroenterology (ACG), and American Gastroenterological Association (AGA) were included for a total of 45 documents. Clinical practice guidelines (CPG) and expert consensus documents (ECD) were analyzed. Document sections that pertained to children or adolescents were excluded.

Cost/value statements were independently abstracted and categorized by two authors (E.A. and A.K). Discrepancies were resolved by consensus and in consultation with the principal investigator (N.U.). Cost/value considerations were classified based on protocols adapted from prior studies.^2, 3^ Each guidance document and guidance statement were evaluated for the presence of cost/value considerations and classified as explicit, implicit, excluded, or unmentioned. Each cost/value statement was subsequently classified into four categories based on how it was used: 1) to emphasize the cost/value economic impact, 2) to advocate for cost/value-related topics, 3) to support specific recommendations or 4) to highlight a gap in cost/value evidence. Cost/value statements that supported specific recommendations were subcategorized as whether their focus was on societal or patient-level costs. Cost/value statements that supported specific recommendations were further subcategorized into five categories based on whether the statement supported or discouraged use of a guidance statement – 1) recommended use given equal effectiveness at lower cost in routine cases; 2) recommended use because the incremental benefit justified the additional cost; 3) recommended use to reduce future costs; 4) discouraged use because the cost-to-benefit ratio was uncertain; 5) discouraged use because incremental benefit did not justify additional costs.

Frequency of inclusion of cost/value statements was expressed as a percentage of all documents. Categorization of cost/value statements were expressed as a percentage of all statements. Comparisons of continuous outcomes were performed using Wilcoxon rank-sum testing. Statistical analyses were performed using STATA software version 16.1 (StataCorp LLC). No patient-level data were accessed to require IRB approval by Massachusetts General Hospital.

**Supplemental Figure 1: Percentage of Cost-Value Statements per Guideline**

**Supplemental Figure 2: Cost/Value Statement Classification Schema**
